# In vivo monoclonal antibody efficacy against SARS-CoV-2 variant strains

**DOI:** 10.21203/rs.3.rs-448370/v1

**Published:** 2021-04-23

**Authors:** Michael Diamond, Rita Chen, Emma Winkler, James Case, Ishmael Aziati, Traci Bricker, Astha Joshi, Tamarand Darling, Baoling Ying, John Errico, Swathi Shrihari, Laura VanBlargan, Xuping Xie, Pavlo Gilchuk, Seth Zost, Lindsay Droit, Zhuoming Liu, Spencer Stumpf, David Wang, Scott Handley, W Stine, Pei-Yong Shi, Miguel Garcia-Knight, Raul Andino, Charles Chiu, Ali Ellebedy, Daved Fremont, Sean Whelan, James Crowe, Lisa Purcell, Davide Corti, Andrianus Boon

**Affiliations:** Washington University School of Medicine; Washington University School of Medicine; Washington University in St. Louis; Washington University School of Medicine; Washington University School of Medicine; Washington University School of Medicine; Washington University School of Medicine; Washington University School of Medicine; Washington University School of Medicine; Washington University; Washington University School of Medicine; Washington University; University of Texas Medical Branch; Vanderbilt University Medical Center; Vanderbilt University Medical Center; Washington University School of Medicine; Washington University School of Medicine; Washington University; Washington University in St. Louis; Washington University; AbbVie; The University of Texas Medical Branch at Galveston; University of California, San Francisco; University of California, San Francisco; University of California, San Francisco; Washington University School of Medicine; Washington University School of Medicine; Washington University in Saint Louis; Vanderbilt University Medical Center; Vir Biotechnology, Washington University School of Medicine; Vir Biotechnology, Washington University School of Medicine; Washington University School of Medicine

**Keywords:** SARS-CoV-2 variant strains, variations, antibodies

## Abstract

Rapidly-emerging variants jeopardize antibody-based countermeasures against SARS-CoV-2. While recent cell culture experiments have demonstrated loss of potency of several anti-spike neutralizing antibodies against SARS-CoV-2 variant strains1-3, the in vivo significance of these results remains uncertain. Here, using a panel of monoclonal antibodies (mAbs) corresponding to many in advanced clinical development by Vir Biotechnology, AbbVie, AstraZeneca, Regeneron, and Lilly we report the impact on protection in animals against authentic SARS-CoV-2 variants including WA1/2020 strains, a B.1.1.7 isolate, and chimeric strains with South African (B.1.351) or Brazilian (B.1.1.28) spike genes. Although some individual mAbs showed reduced or abrogated neutralizing activity against B.1.351 and B.1.1.28 viruses with E484K spike protein mutations in cell culture, low prophylactic doses of mAb combinations protected against infection in K18-hACE2 transgenic mice, 129S2 immunocompetent mice, and hamsters without emergence of resistance. Two exceptions were mAb LY-CoV555 monotherapy which lost all protective activity in vivo, and AbbVie 2B04/47D11, which showed partial loss of activity. When administered after infection as therapy, higher doses of mAb cocktails protected in vivo against viruses displaying a B.1.351 spike gene. Thus, many, but not all, of the antibody products with Emergency Use Authorization should retain substantial efficacy against the prevailing SARS-CoV-2 variant strains.

## Introduction

Severe acute respiratory syndrome coronavirus 2 (SARS-CoV-2) has caused the global coronavirus disease 2019 (COVID-19) pandemic and resulted in more than 140 million confirmed infections and over 3 million deaths. The sustained nature of the COVID-19 pandemic and its accompanying extensive morbidity and mortality have made the development and immediate deployment of therapeutics and vaccines an urgent global health priority^4^. Indeed, the emergency use authorization (EUA) of several monoclonal antibody (mAb) therapies and mRNA, inactivated, and viral-vectored vaccines has provided hope for controlling infection and curtailing the pandemic.

Currently authorized antibody countermeasures against SARS-CoV-2 target the spike protein from strains circulating during the early phases of the pandemic in 2020. The SARS-CoV-2 spike protein binds the cell-surface receptor angiotensin-converting enzyme 2 (ACE2) to facilitate viral entry into and infection of human cells^5^. Upon cell attachment, SARS-CoV-2 spike proteins are cleaved by host proteases into S1 and S2 fragments. The S1 protein includes the N-terminal (NTD) and receptor binding (RBD) domains, whereas the S2 protein promotes membrane fusion. The RBD, in particular, is the target of many potently neutralizing monoclonal^6-10^ and serum polyclonal antibodies^11^.

Over the past several months, SARS-CoV-2 variant strains have emerged in the United Kingdom (B.1.1.7), South Africa (B.1.351), Brazil (B.1.1.28 [also called P.1]) and elsewhere containing substitutions in the spike protein in the NTD and the receptor binding motif (RBM) of the RBD. Experiments with pseudoviruses and authentic infectious SARS-CoV-2 strains suggest that neutralization by a substantial fraction of previously generated antibodies may be diminished against variants expressing mutations in the spike gene, especially at position E484^1-3,12,13^. However, the *in vivo* significance of this loss of mAb neutralizing activity remains uncertain, particularly for combination mAb therapies, as high doses could compensate for changes in neutralization potency. Here, using mice and hamsters, we assessed the protective activity of clinically relevant mAbs against WA1/2020 strains and a panel of SARS-CoV-2 variants including a B.1.1.7 isolate, and chimeric strains with South African (B.1.351) or Brazilian (B.1.1.28) spike genes. We tested cocktails of mAbs from AbbVie (2B04/47D11) and Vir Biotechnology (S309/S2E12) as well as ones corresponding to those from AstraZeneca (COV2-2130/COV2-2196), Regeneron (REGN10933/REGN10987), and Lilly (LY-CoV555) as prophylaxis or therapy against SARS-CoV-2 in K18-hACE2 transgenic mice, 129S2 immunocompetent mice, and Syrian hamsters. Whereas several antibody combinations conferred protection in both mouse models with all variant strains tested, the 2B04/47D11 combination and LY-CoV555 showed reduced or complete loss of protective activity. One of the combinations (COV2-2130/COV2-2196) also showed equivalent protective activity in hamsters against WA1/2020 D614G and the chimeric strain with a B.1.351 spike.

## Results

**Figure 1:**
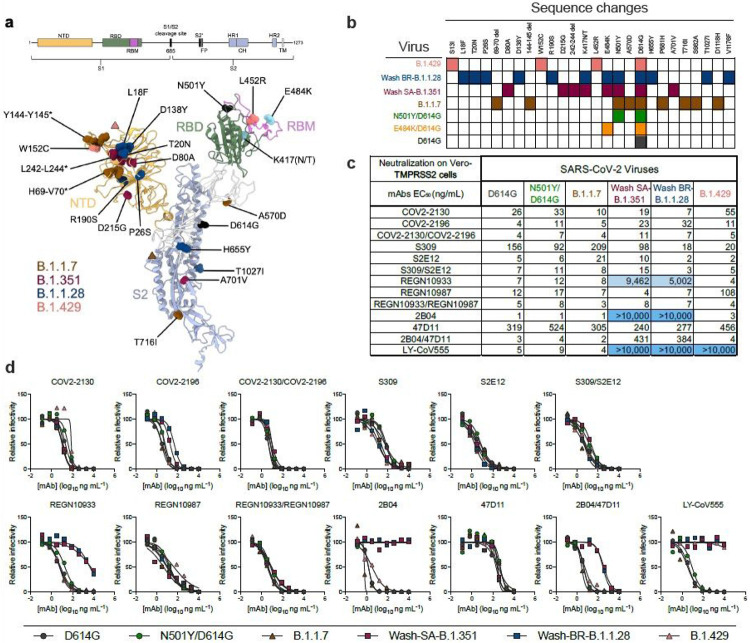
Neutralization of SARS-CoV-2 variant strains by clinically relevant mAbs. (a) SARS-CoV-2 variant substitutions mapped onto the structure of the spike protein. Schematic layout of the spike protein monomer is depicted at the top. Structure of spike monomer (PDB: 7C2L with RBD from PDB: 6W41) is depicted as a cartoon, with NTD, RBD, RBM, and S2 colored orange, green, magenta, and light blue, respectively. Substitutions for each variant (B.1.1.7: 69-70 deletion, 144-145 deletion, N501Y, A570D, D614G, P681H,and T716I; B.1.351: 242-244 deletion, D80A, D215G, K417N, E484K, N501Y, D614G, and A701V; B.1.1.28: L18F, T20N, P26S, D138Y, R190S, K417T, E484K, N501Y, D614G, H655Y, T1027I; B.1.429: S13I, W152C, L452R) are shown as spheres and colored accordingly. Substitutions shown in cyan (E484K and K417[N/T]) are shared by B.1.351 and B.1.1.28. Substitutions shown in black (D614G and N501Y) are shared by B.1.1.7, B.1.351, and B.1.1.28. Pink and brown triangles show approximate locations of S13 and P681, which were not modelled in the original structures. Structural figure generated using UCSF ChimeraX45. (b) Viruses used with indicated mutations in the spike protein. (c) Summary of EC50 values (ng/mL) of neutralization of SARS-CoV-2 viruses performed in Vero-TMPRSS2 cells. Blue shading of cells indicates a partial (EC50 > 1,000 ng/mL) or complete (EC50 > 10,000 ng/mL) loss of neutralizing activity. (d) Neutralization curves comparing the sensitivity of SARS-CoV-2 strains to the indicated individual or combinations of mAbs. Data are representative of two to five experiments, each performed in technical duplicate.

To evaluate the effects of SARS-CoV-2 strain variation on mAb protection, we assembled a panel of infectious SARS-CoV-2 strains with sequence substitutions in the spike gene (Fig 1a-b). A B.1.1.7 isolate from the United Kingdom had signature changes in the spike gene^14^ including the 69-70 and 144-145 deletions, and N501Y, A570D, D614G, and P681H substitutions. A B.1.429 isolate from California contained the characteristic S13I, W152C, and L452R changes. We also used a previously generated Washington SARS-CoV-2 strain with a D614G substitution (WA1/2020 D614G), a SARS-CoV-2 strain with N501Y and D614G substitutions (WA1/2020 N501Y/D614G), and recombinant, chimeric SARS-CoV-2 strains with a South African (Wash SA-B.1.351; D80A, D215G, 242-244 deletion, K417N, E484K, N501Y, D614G, and A701V) or Brazilian (Wash BR-B.1.1.28; L18F, T20N, P26S, D138Y, R190S, K417T, E484K, N501Y, D614G, H655Y, T1027I, and V1176F) spike genes in the Washington strain background^1,15^. All viruses were propagated in Vero cells expressing transmembrane protease serine 2 (TMPRSS2) to prevent the emergence of mutations at or near the furin cleavage site in the spike protein, which occurs with passage in Vero E6 cells^16^ and can impact virulence^17^. All viruses were deep-sequenced to confirm the presence of expected mutations prior to use *in vitro* or *in vivo* (Supplementary Table S1).

We first assessed the impact of SARS-CoV-2 spike variation on antibody neutralization in Vero-TMPRSS2 cells (Fig 1c-d) using the WA1/2020 D614G, WA1/2020 N501Y/D614G, B.1.1.7, Wash SA-B.1.351, Wash BR-B.1.1.28, and B.1.429 viruses. We tested individual and cocktails of mAbs in clinical development that target the RBD including 2B04/47D11 (AbbVie), S309/S2E12 (Vir Biotechnology), COV2-2130/COV2-2196 (Vanderbilt University Medical Center with engineered derivatives being evaluated by AstraZeneca), REGN10933/REGN10987 (synthesized based on casirivimab and imdevimab sequences from Regeneron), and LY-CoV555 (synthesized based on bamlanivimab sequences from Lilly). All individual mAbs tested efficiently neutralized the WA1/2020 D614G, WA1/2020 N501Y/D614G, and B.1.1.7 strains, and several mAbs (COV2-2130, COV2-2196, S309, S2E12, and 47D11) showed little change in potency against the Wash SA-B.1.351, Wash BR-B.1.1.28, and B.1.429 strains (Fig 1c-d). In comparison, REGN10987 or LY-CoV555 respectively showed a ~10-fold or complete loss in inhibitory activity against the B.1.429 strain, which is consistent with studies identifying L452 and adjacent residues as interaction sites for these mAbs (Table 1). Moreover, REGN10933, LY-CoV555, and 2B04 exhibited a marked loss or complete absence of neutralizing activity against Wash SA-B.1.351, Wash BR-B.1.1.28, and viruses containing the E484K mutation (Fig 1c-d and Extended Data Fig 1), which corresponds with structural and mapping studies (Table 1). Analysis of mAb cocktails showed that COV2-2130/COV2-2196, S309/S2E12, and REGN10933/REGN10987 neutralized all virus strains tested, with the latter combination retaining potency corresponding to the mAb with inhibitory activity in the cocktail for a given virus. In comparison, while the 2B04/47D11 mAb combination efficiently neutralized WA1/2020 D614G, WA1/2020 N501Y/D614G, B.1.1.7, and B.1.429 strains, its inhibitory activity against Wash SA-B.1.351 and Wash BR-B.1.1.28 reflected the less potent 47D11 mAb component (EC_50_ of 384-431 ng/mL) (Fig 1c-d).

To evaluate the efficacy of the mAb combinations *in vivo*, we initially used the K18-hACE2 transgenic mouse model of SARS-CoV-2 pathogenesis in which human ACE2 expression is driven by the cytokeratin-18 gene promoter^18-19^. In prior studies, we established that low (2 mg/kg) doses of several different anti-RBD neutralizing human mAbs provide a threshold of protection against the WA1/2020 strain when administered as prophylaxis^20^. Accordingly, we gave K18-hACE2 mice a single 40 μg (~2 mg/kg total) dose of mAb combinations (2B04/47D11, S309/S2E12, COV2-2130/COV2-2196, or REGN10933/REGN10987) or LY-CoV555 as monotherapy by intraperitoneal injection one day prior to intranasal inoculation with SARS-CoV-2 (10^3^ focus-forming units [FFU] of WA1/2020 N501Y/D614G, B.1.1.7, Wash SA-B.1.351 or Wash BR-B.1.1.28). For these *in vivo* studies, we used a recombinant version of WA1/2020 that encodes N501Y for direct comparison to B.1.1.7, Wash SA-B.1.351 or Wash BR-B.1.1.28, all of which naturally contain this residue. This substitution increases infection and pathogenicity in mice^21,22^ yet did not substantively impact neutralization of the mAbs we tested (Fig 1c). We monitored weight change for six days, and then euthanized animals and harvested tissues for virological and immunological analyses.

**Figure 2:**
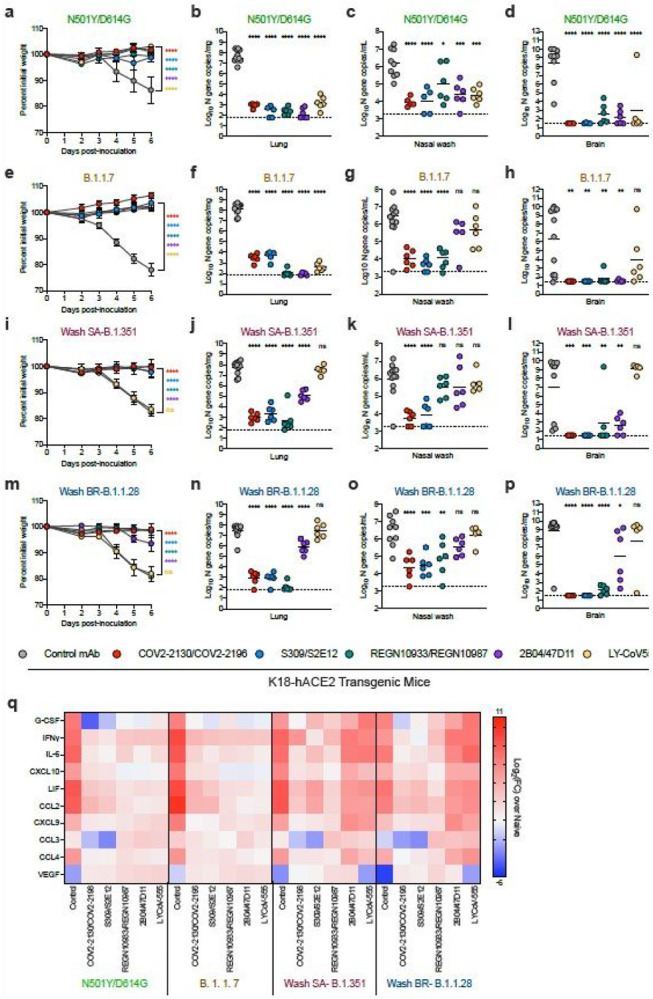
Antibody prophylaxis against SARS-CoV-2 variants in K18-hACE2 mice. (a-q) 8-10-week-old female and male K18-hACE2 transgenic mice received 40 g (~2 mg/kg) of the indicated mAb treatment by intraperitoneal injection one day before intranasal inoculation with 103 FFU of the indicated SARS-CoV-2 strain. Tissues were collected at 6 dpi. (a, e, i, m) Weight change following infection with SARS-CoV-2 (mean ± SEM; n = 6-12 mice per group, two experiments; one-way ANOVA with Dunnett’s test of area under the curve: ns, not significant, **** P < 0.0001). Viral RNA levels in the lung (b, f, j, n), nasal washes (c, g, k, o), and brain (d, h, l, p) were measured (n = 6-12 mice per group, two experiments; one-way ANOVA with Dunnett’s test with comparison to control mAb: ns, not significant, * P < 0.05, ** P < 0.01, *** P < 0.001, **** P < 0.0001). Dotted line indicates the limit of detection of the assay. Heat map of cytokine and chemokine protein expression levels in lung homogenates collected at 6 dpi from the indicated groups (q). Data are presented as log2 fold-change over naive animals. Blue, reduction; red, increase.

Compared to a control human mAb (anti-West Nile virus hE16^23^), a single 40 mg prophylaxis dose of the anti-SARS-CoV-2 mAbs conferred substantial protection against WA1/2020 N501Y/D614G-induced weight loss and viral burden in the lungs, nasal washes, brain, spleen, and heart in the K18-hACE2 mice at 6 days post-infection (dpi) (Fig 2a-d, Extended Data Fig 2 and 3a). While all of the anti-SARS-CoV-2 mAb cocktails conferred protection against weight loss caused by B.1.1.7, Wash SA-B.1.351 or Wash BR-B.1.1.28, LY-CoV555 monotherapy protected only against the B.1.1.7 strain (Fig 2e, i, and m). Some of the antibodies provided less virological protection against the B.1.1.7, Wash SA-B.1.351 or Wash BR-B.1.1.28 strains in specific tissues. Whereas all mAb groups protected against B.1.1.7 infection in the lung, 2B04/47D11 and LY-CoV555 failed to perform as well in nasal washes, and LY-CoV555 showed reduced protection against infection in the brain (Fig 2f-h). Sanger sequencing analysis of the RBD region of viral RNA of brain, nasal wash, and lung samples from animals treated with these mAbs did not show evidence of neutralization escape (Supplementary Table S2). 2B04/47D11 and LY-CoV555-treated animals also showed greater virus breakthrough than the other tested antibodies when challenged with Wash SA-B. 1.351 or Wash BR-B.1.1.28 viruses: 2B04/47D11 reduced viral burden in the lungs, nasal washes, and brain (500-10,000-fold) much less efficiently than other mAb cocktails, and LY-CoV555 mAb treatment conferred virtually no virological protection in any tissue analyzed (Fig 2j-l and n-p and Extended Data Fig 3b). Compared to the COV2-2130/COV2-2196 and S309/S2E12 combinations, REGN10933/REGN10987 also showed less ability to reduce viral RNA levels in nasal washes of K18-hACE2 mice infected with Wash SA-B.1.351 or Wash BR-B.1.1.28 viruses.

An excessive pro-inflammatory host response to SARS-CoV-2 infection is hypothesized to contribute to pulmonary pathology and severe COVID-19^24^. To evaluate further the extent of protection conferred by the different mAb groups against the SARS-COV-2 variant viruses, we measured pro-inflammatory cytokine and chemokines in lung homogenates harvested at 6 dpi (Fig 2q and Extended Data Fig 4). This analysis showed a strong correspondence with viral RNA levels in the lung: (a) compared to the control mAb, S309/S2E12, COV2-2130/COV2-2196, and REGN10933/REGN10987 combinations showed markedly reduced levels of pro-inflammatory cytokines and chemokines (G-CSF, IFN-g, IL-6, CXCL10, LIF, CCL2, CXCL9, CCL3, and CCL4) after infection with WA1/2020 N501Y/D614G, B.1.1.7, Wash SA-B.1.351 or Wash BR-B.1.1.28; (b) prophylaxis with 2B04/47D11 or LY-CoV555 resulted in reduced inflammatory cytokine and chemokine levels in mice infected with WA1/2020 N501Y/D614G and B.1.1.7, with substantially less improvement in animals infected with Wash SA-B.1.351 and Wash BR-B.1.1.28.

Given that a 40 mg dose of S309/S2E12, COV2-2130/COV2-2196, and REGN10933/REGN10987 combinations prevented infection and inflammation caused by the different SARS-CoV-2 strains, we next tested a ten-fold lower 4 mg dose (~0.2 mg/kg) to assess for possible differences in protection. Prophylaxis with COV2-2130/COV2-2196, S309/S2E12, REGN10933/REGN10987, or 2B04/47D11 protected K18-hACE2 mice against weight loss caused by all four viruses (Extended Data Fig 5a-d). Whereas the COV2-2130/COV2-2196, S309/S2E12, and REGN10933/REGN10987 mAb combinations reduced viral RNA levels in the lung at 6 dpi in K18-hACE2 mice infected with WA1/2020 N501Y/D614G, B.1.1.7, Wash SA-B.1.351, or Wash BR-B.1.1.28, the 2B04/47D11 treatment conferred protection against B.1.1.7 and WA1/2020 N501Y/D614G but not against Wash SA-B. 1.351 and Wash BR-B.1.1.28 viruses at this lower dose (Extended Data Fig 5e-h). In comparison, in nasal washes, all four mAb cocktails resulted in relatively similar reductions in viral RNA levels at 6 dpi of animals inoculated with WA1/2020 N501Y/D614G, B.1.1.7, Wash SA-B. 1.351 or Wash BR-B.1.1.28 (Extended Data Fig 5i-l). Even at this low treatment dose, with the exception of some substantive breakthrough events (>6 log 10 copies of N/mg: COV2-2130/COV2-2196 [2 of 24 mice];S309/S2E12 [6 of 24 mice]; REGN10933/REGN10987 [1 of 24 mice]; and 2B04/47D11 [6 of 24 mice]), the mAb combinations generally prevented viral dissemination to and high-level infection of the brain (Extended Data Fig 5m-p and Supplementary Table S2).

**Figure 3:**
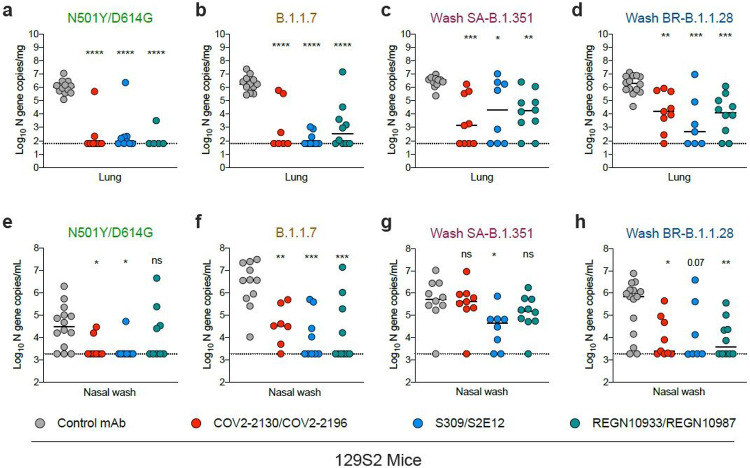
Antibody-mediated protection against SARS-CoV-2 variants in 129S2 mice. 6-7-week-old female and male immunocompetent 129S2 mice received 40 mg (~ 2 mg/kg) of the indicated mAb treatment by intraperitoneal injection one day before intranasal inoculation with 103 FFU of WA1/2020 N501Y/D614G, Wash SA-B.1.351, or Wash BR-B.1.1.28 and 105 FFU of B.1.1.7. Tissues were collected at 3 dpi. Viral RNA levels in the lung (a-d) or nasal washes (e-h) were determined (n = 7-12 mice per group, pooled from two experiments; one-way ANOVA with Dunnett’s test with comparison to control mAb: ns, not significant, * P < 0.05, ** P < 0.01, *** P < 0.001). Dotted line indicates the limit of detection of the assay.

Although K18-hACE2 mice have been used extensively to test vaccines and therapeutics against SARS-CoV-2^20,25-28^, the high level and distinct pattern of transgene expression in these animals could impact entry pathways, and neutralization and protection conferred by anti-RBD antibodies. As an alternative model for evaluating mAb efficacy, we tested immunocompetent, inbred 129S2 mice, which are permissive to infection by SARS-CoV-2 strains encoding an N501Y substitution without ectopic hACE2 expression^21,22^; presumably, the N501Y adaptive mutation enables efficient engagement of murine (m)ACE2. We administered a single 40 μg (~2 mg/kg) dose of mAb cocktails (COV2-2130/COV2-2196, S309/S2E12, or REGN10933/REGN10987) or a control mAb via intraperitoneal injection one day prior to intranasal inoculation with 10^3^ FFU of WA1/2020 N501Y/D614G, Wash SA-B.1.351, or Wash BR-B.1.1.28, and 10^5^ FFU of B.1.1.7 (Fig 3). A higher inoculating dose of B.1.1.7 was required to obtain equivalent levels of viral RNA in the lung compared to the other three viruses. At 3 dpi, we harvested tissues for viral burden analyses; at this time point, reproducible weight loss was not observed. All three mAb cocktails tested (COV2-2130/COV2-2196, S309/S2E12, and REGN10933/REGN10987) protected 129S2 mice against infection in the lung by all SARS-CoV-2 strains as judged by reductions in viral RNA levels (Fig 3a-d); despite some variability, we observed a trend toward less complete protection in animals infected with Wash SA-B.1.351 and Wash BR-B.1.1.28 strains (Fig 3c-d and Extended Data Fig 3c-f). When we evaluated the nasal washes, reductions in viral RNA levels were diminished with the Wash SA-B.1.351 virus, especially for the COV2-2130/COV2-2196 and REGN10933/REGN10987 combinations (Fig 3e-h). Sequencing analysis of lung samples from the infected 129S2 mice also did not reveal evidence of acquisition of mutations in the RBD (Supplementary Table S2).

**Figure 4:**
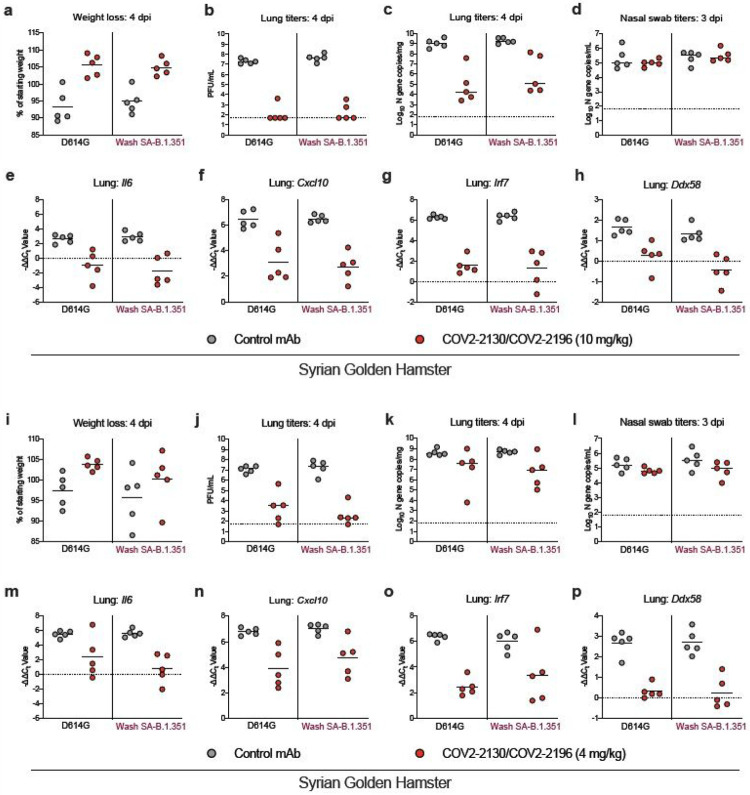
COV2-2130/COV2-2196 antibody cocktail protects hamsters against historical and variant SARS-CoV-2 strains. Six-week-old male Syrian golden hamsters received a single 800 mg (~10 mg/kg) (a-h) or 320 mg (~4 mg/kg) dose (i-p) of COV2-2130/COV2196 mAb cocktail or control mAb by intraperitoneal injection one day before intranasal inoculation with 5 x 105 FFU of WA1/2020 D614G or Wash SA-B.1.351 viruses. Nasal swabs and lung tissues were collected at 3 and 4 dpi, respectively. (a, i) Weight change following infection with SARS-CoV-2 (mean ± SEM; n = 5 animals per group, one experiment). Infectious virus in the lung (b, j) or viral RNA levels in the lung (c, k) and nasal swabs (d, l) were determined (n = 5 animals per group, one experiment). Dotted line indicates the limit of detection of the assay. (e-h, m-p) Cytokine and inflammatory gene expression in lung homogenates collected at 6 dpi from indicated groups. Values were calculated using the DDCt method compared to a naive control group. Because data were obtained from a single experiment (even with multiple animals), statistical analysis was not performed.

The immunocompetent Syrian golden hamster also has been used to evaluate mAb activity against SARS-CoV-2 infection in the upper and lower respiratory tracts^29,30^. We used this animal model to assess independently the inhibitory activity and possible emergence of resistance of one of the mAb combinations (COV2-2130/COV2-2196) against viruses containing the B. 1.351 spike protein at threshold doses of protection. One day prior to intranasal inoculation with 5 x 10^5^ FFU of Wash SA-B.1.351 or WA1/2020 D614G, we treated hamsters with a single 800 μg (~10 mg/kg) or 320 μg (~4 mg/kg) dose of the COV2-2130/COV2-2196 cocktail or isotype control mAb by intraperitoneal injection (Fig 4). Weights were followed for 4 days, and then tissues were harvested for virological and cytokine analysis. At the 800 μg mAb cocktail dose, hamsters treated with COV2-2130/COV2-2196 and infected with WA1/2020 D614G or Wash SA-B.1.351 showed protection against weight loss (Fig 4a) and reduced viral burden levels in the lungs but not nasal swabs compared to the isotype control mAb (Fig 4b-d). Correspondingly, RT-qPCR analysis of a previously described set of cytokines and inflammatory genes^20^ showed reduced mRNA expression in the lungs of hamsters treated with COV2-2130/COV2-2196 (Fig 4e-h). Consensus sequencing of the RBD region of viral RNA samples from the lungs of hamsters treated with COV2-2130/COV2-2196 and inoculated with WA1/2020 D614G or Wash SA-B.1.351 did not show evidence of mutation or escape (Supplementary Table S2). When the lower 320 μg dose of COV2-2130/COV2-2196 was administered, we observed a trend toward protection against weight loss in hamsters infected with WA1/2020 D614G and Wash SA-B.1.351 (Fig 4i). Consistent with a partially protective phenotype, hamsters treated with the lower 320 μg dose of COV2-2130/COV2-2196 and inoculated with either WA1/2020 D614G and Wash SA-B.1.351 showed a trend towards reduced viral RNA in the lungs at 4 dpi and markedly diminished (~10^4^ to 10^5^-fold) levels of infectious virus as determined by plaque assay (Fig 4j-k). The reduction in lung viral load conferred by the lower dose COV2-2130/COV2-2196 corresponded with diminished inflammatory gene expression after infection with either WA1/2020 D614G or Wash SA-B.1.351 (Fig 4m-p). In contrast to the protection seen in the lung, differences in viral RNA were not observed in nasal washes between COV2-2130/COV2-2196 and isotype control mAb-treated animals regardless of the infecting strain (Fig 4l). Sequencing of the RBD of viral RNA from the lungs of COV2-2130/COV2-2196 or isotype mAb-treated hamsters also did not detect evidence of escape mutation selection after infection with WA1/2020 D614G or Wash SA-B.1.351 (Supplementary Table S2). Overall, these studies in hamsters with near threshold dosing of the COV2-2130/COV2-2196 mAb cocktail establish equivalent protection and an absence of rapid escape against SARS-CoV-2 containing spike proteins from historical or variant strains.

**Figure 5:**
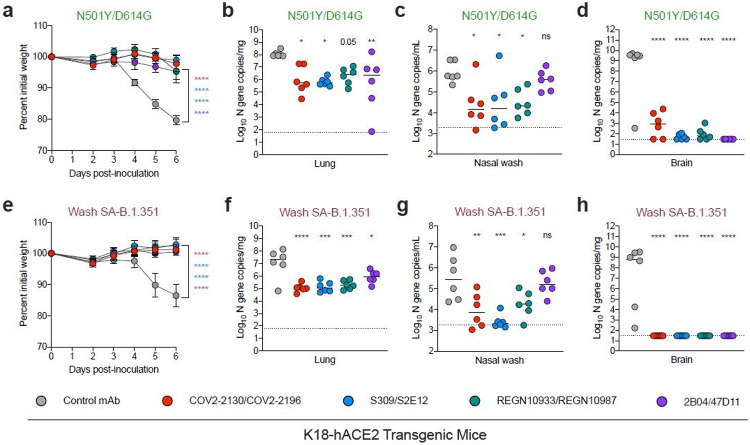
Post-exposure antibody therapy against SARS-CoV-2 variants in K18-hACE2 mice. (a-h) 8-10-week-old female and male K18-hACE2 transgenic mice were administered 103 FFU of the indicated SARS-CoV-2 strain by intranasal inoculation. One day later, mice received 200 mg (~10 mg/kg) of the indicated mAb treatment by intraperitoneal injection. Tissues were collected at 6 dpi. (a, e) Weight change following infection with SARS-CoV-2 (mean ± SEM; n = 6 mice per group, two experiments; one-way ANOVA with Dunnett’s test of area under the curve: **** P < 0.0001). Viral RNA levels in the lung (b, f), nasal wash (c, g), and brain (d, h) (n = 6 mice per group, two experiments; one-way ANOVA with Dunnett’s test with comparison to control mAb: ns, not significant, * P < 0.05, ** P < 0.01, *** P < 0.001, **** P < 0.0001). Dotted line indicates the limit of detection of the assay.

As mAbs are being developed clinically as therapeutics, we assessed their post-exposure efficacy against the SARS-CoV-2 strain expressing the B.1.351 spike protein using the stringent K18-hACE2 model. We administered a single, higher 200 μg (~10 mg/kg) dose of COV2-2130/COV2-2196, S309/S2E12, REGN10933/REGN10987 or 2B04/47D11 by intraperitoneal injection one day after inoculation with 10^3^ FFU of WA1/2020 N501Y/D614G or Wash SA-B.1.351, and then monitored the mice for six days prior to necropsy and virological analysis (Fig 5). We did not test the LY-CoV555 mAb in these therapeutic experiments, since it failed to protect against Wash SA-B.1.351 as prophylaxis. Compared to the control mAb-treated animals, which lost at least 15% of their starting weight over the 6 days of the experiment, each of the mAb cocktails prevented weight loss induced by WA1/2020 N501Y/D614G or Wash SA-B.1.351 infection (Fig 5a and e). COV2-2130/COV2-2196, S309/S2E12, and REGN10933/REGN10987 mAb cocktail treatments resulted in reduced infectious virus and viral RNA levels in lung homogenates, and viral RNA levels in nasal washes and brain homogenates from animals infected with either WA1/2020 N501Y/D614G or Wash SA-B.1.351 (Fig 5b-d, f-h and Extended Data Fig 3g-h). In comparison, while the 2B04/47D11 mAb cocktail reduced viral RNA levels in the lungs, it showed less protection in the nasal washes of WA1/2020 N501Y/D614G and Wash SA-B.1.351 infected mice.

## Discussion

With the emergence of several SARS-CoV-2 variants, it remains uncertain whether currently developed vaccines and antibody-based therapies will lose efficacy^31^. Many of the mutations and deletions in the spike proteins of variant strains occur in the N-terminal domain and the RBD, including within or proximal to the hACE2 receptor binding motif. Cell-culture based studies have shown that several of these mutations, especially those at positions 452 and 484, reduce neutralization capacity of monoclonal and serum antibodies derived from naturally infected or vaccinated individuals^1-3,32,33^. Here we evaluated antibodies forming the basis of five different mAb therapies in clinical development for *in vivo* efficacy against infection by SARS-CoV-2 variants including a B.1.1.7 isolate and chimeric strains with B.1.351 or B.1.1.28 spike genes. Monotherapy with LY-CoV555, an antibody corresponding to *bamlanivimab*^34^, *showed complete neutralization escape in cell culture and failed to confer any protection against viruses containing E484K substitutions. In contrast, all cocktails of two neutralizing mAbs conferred protection to varying degrees even if one of the constituent mAbs showed reduced activity due to resistance. Moreover, the higher doses of mAbs used in patients (e.g., 2.4 g or ~ 35 mg/kg for* casirivimab and imdevimab [REGN mAbs]) could compensate for loss in neutralization potency.

Combination therapy with multiple mAbs in our study (COV2-2130/COV2-2196, S309/S2E12, REGN10933/REGN10987, or 2B04/47D11) was protective in mice and hamsters against the variant strains, highlighting the importance of using multiple mAbs recognizing distinct epitopes rather than monotherapy to control SARS-CoV-2 infection. Indeed, the emergency use authorization for *bamlanivimab* (LY-CoV555) as monotherapy recently was revoked, since the antibody does not efficiently reduce SARS-CoV-2 infection of several variants of concern that are spreading globally^35^; instead, a combination of *bamlanivimab and* etesevimab is now recommended even though some strains containing E484 and K417 mutations (*e.g.,* B. 1.351 and B.1.1.28) likely will have resistance to *both mAb components (*^2,36^ *and* Extended Data Fig 6*). in our study combination* therapy with two mAbs including one (2B04) that failed to neutralize a virus containing the E484K mutation still protected when administered at higher doses, although the reduction in viral burden was less than with other mAb cocktails at equivalent doses. Beyond a loss of potency against already circulating resistant variants, antibody monotherapy can be compromised within an individual by rapid selection of escape mutations *de novo* or enrichment of pre-existing mutants in the quasispecies present at low frequency. Consistent with this idea, in other animal experiments with SARS-CoV-2, we have observed the rapid emergence of resistance against antibody monotherapy, resulting in the accumulation of mutations at RBD residues 476, 477 484, and 487, only some of which were detectable in our parental virus stocks by next generation sequencing (^37^ and M. Diamond, A. Boon, and A. Ellebedy, unpublished data). Remarkably, and despite amplifying the RBD sequence from 96 brain, nasal wash, and lung samples from mice and hamsters treated with the different mAb combinations, we did not detect a single escape mutant. Although further study is warranted, combination mAb treatment may prevent escape through synergistic interactions *in vivo* or by driving selection of mutants with compromised fitness.

At the lower doses of mAbs tested, we observed some differences in mAb cocktail efficacy between rodent models, which could be due to host variation, viral variation, or both. For example, mutations in the RBD can affect mAb binding as well as ACE2 binding^38^. Mutation at position 501 of the spike is of particular interest, since it enables mouse adaptation^21,22^ and is present in many variants of concern (*e.g*., B.1.1.7, B.1.351, and B.1.1.28). The N501Y change associated with infection of conventional laboratory mice could facilitate virus engagement with murine ACE2 or possibly other putative target receptors^39^. Beyond this, polymorphisms in or differences of expression of host receptors on key target cells also could impact SARS-CoV-2 infection in different hosts and the inhibitory effects of neutralizing antibodies. As both viral and host sequences determine the interface between SARS-CoV-2 spike and its cell entry receptors like ACE2, mAb interactions and potency could be affected in different species of animals. Changes in the affinity of interaction between spike proteins and receptors can impact the stoichiometry of neutralizing antibody binding required to inhibit infection^40^. Although further study of antibody-based countermeasures *in vivo* is required, the complexity of antibody-spike protein-receptor interactions likely explains some of the variation in protection K18-hACE2 mice, 129S2 mice, and hamsters. Alternatively, the pharmacokinetics and/or biodistribution of antibodies in these animals also could vary and affect efficacy. In the animal models we tested, we did not observe marked differences in serum antibody levels in the context of viral challenge (Supplementary Table S3).

Cell culture-based analyses of individual neutralizing antibodies in clinical development with pseudoviruses and authentic SARS-CoV-2 containing substitutions corresponding to those in circulating variants suggested that adjustments to therapeutic antibody regimens might be necessary to maintain efficacy^1,2,41-43^. Although our *in vivo* studies with several SARS-CoV-2 variant strains in multiple rodent models suggest this conclusion likely holds for mAb monotherapy, four different mAb combinations performed remarkably well even when a particular variant containing an E484K mutation was fully resistant to one mAb component, as rapid escape over the short time course of study was not observed in nasal washes or lung tissues. While corroborative analysis of antibody efficacy in non-human primates and humans is needed, especially under conditions of protracted infection or high viral burden, our results suggest that, as described previously with the historical WA1/2020 strain^44^, combination therapy with neutralizing mAbs may retain efficacy against emerging SARS-CoV-2 variants and limit the development of resistance.

## Methods

### Cells.

Vero-TMPRSS2 cells^46^ and Vero-hACE2-TMPRSS2 (gift of A. Creanga and B. Graham, NIH, Bethesda, MD) cells were cultured at 37°C in Dulbecco’s Modified Eagle medium (DMEM) supplemented with 10% fetal bovine serum (FBS), 10 mM HEPES pH 7.3, 1 mM sodium pyruvate, 1× non-essential amino acids, and 100 U/ml of penicillin–streptomycin. Vero-TMPRSS2 cells were supplemented with 5 mg/mL of blasticidin and Vero-hACE2-TMPRSS2 cells were supplemented with 10 μg/mL of puromycin.

### Viruses.

The WA1/2020 recombinant strain with substitutions (D614G or N501Y/D614G) were obtained from an infectious cDNA clone of the 2019n-CoV/USA_WA1/2020 strain as described previously^47^. The South African (B. 1.351) and Brazilian (B. 1.1.28) variant spike genes were introduced into the WA1/2020 backbone as described previously^1^. The B.1.1.7 and B.1.429 isolates were obtained from nasopharyngeal isolates. All viruses were passaged once in Vero-TMPRSS2 cells and subjected to next-generation sequencing as described previously^1^ to confirm the introduction and stability of substitutions (Supplementary Table S1). All virus experiments were performed in an approved biosafety level 3 (BSL-3) facility.

### Monoclonal antibody purification.

The mAbs studied in this paper (COV2-2196, COV2-2130, S309, S2E12, 2B04, 47D11, REGN10933, REGN10987, LY-CoV555, and CB6) have been described previously^6,8,10,44,48-51^. COV2-2196 and COV2-2130 mAbs were produced after transient transfection using the Gibco ExpiCHO Expression System (ThermoFisher Scientific) following the manufacturer’s protocol. Culture supernatants were purified using HiTrap MabSelect SuRe columns (Cytiva, formerly GE Healthcare Life Sciences) on an AKTA Pure chromatographer (GE Healthcare Life Sciences). Purified mAbs were buffer-exchanged into PBS, concentrated using Amicon Ultra-4 50-kDa centrifugal filter units (Millipore Sigma) and stored at −80 °C until use. Purified mAbs were tested for endotoxin levels (found to be less than 30 EU per mg IgG). Endotoxin testing was performed using the PTS201F cartridge (Charles River), with a sensitivity range from 10 to 0.1 EU per mL, and an Endosafe Nexgen-MCS instrument (Charles River). S309, S2E12, REGN10933, REGN10987, CB6, and LY-CoV555 mAb proteins were produced in CHOEXPI cells and affinity purified using HiTrap Protein A columns (GE Healthcare, HiTrap mAb select Xtra #28-4082-61). Purified mAbs were suspended into 20 mM histidine, 8% sucrose, pH 6.0. The final products were sterilized by filtration through 0.22μm filters and stored at 4°C.

### Mouse experiments.

Animal studies were carried out in accordance with the recommendations in the Guide for the Care and Use of Laboratory Animals of the National Institutes of Health. The protocols were approved by the Institutional Animal Care and Use Committee at the Washington University School of Medicine (assurance number A3381–01). Virus inoculations were performed under anesthesia that was induced and maintained with ketamine hydrochloride and xylazine, and all efforts were made to minimize animal suffering.

Heterozygous K18-hACE2 C57BL/6J mice (strain: 2B6.Cg-Tg(K18-ACE2)2Prlmn/J) and 129 mice (strain: 129S2/SvPasCrl) were obtained from The Jackson Laboratory and Charles River Laboratories, respectively. Animals were housed in groups and fed standard chow diets. Six- to ten-week-old mice of both sexes were administered 10^3^ or 10^5^ FFU of the respective SARS-CoV-2 strain by intranasal administration.

For antibody prophylaxis and therapeutic experiments, animals were administered the indicated mAb dose by intraperitoneal injection one day before or after intranasal inoculation with the indicated SARS-CoV-2 strain.

### Hamster experiments.

Six-month-old male Syrian hamsters were purchased from Charles River Laboratories and housed in microisolator units. All hamsters were allowed free access to food and water and cared for under United States Department of Agriculture (USDA) guidelines for laboratory animals. Hamsters were administered by intraperitoneal injection mAbs COV2-2130 + COV2-2196 or isotype control (4 or 10 mg/kg depending on the experiment). One day later, hamsters were given 5 x 10^5^ FFU of SARS-CoV-2 (2019-nCoV/USA-WA1/2020) by the intranasal route in a final volume of 100 μL. All hamsters were monitored for body weight loss until humanely euthanized at 4 dpi. Nasal swabs were collected 3 dpi. All procedures were approved by the Washington University School of Medicine (assurance number A3381–01). Virus inoculations and antibody transfers were performed under anesthesia that was induced and maintained with 5% isoflurane. All efforts were made to minimize animal suffering.

### Focus reduction neutralization test.

Serial dilutions of mAbs (starting at 10 mg/mL dilution) were incubated with 10^2^ focus-forming units (FFU) of different strains or variants of SARS-CoV-2 for 1 h at 37°C. Antibody-virus complexes were added to Vero-TMPRSS2 or Vero-hACE2-TMPRSS2 cell monolayers in 96-well plates and incubated at 37°C for 1 h. Subsequently, cells were overlaid with 1% (w/v) methylcellulose in MEM supplemented with 2% FBS. Plates were harvested 24 h later by removing overlays and fixed with 4% PFA in PBS for 20 min at room temperature. Plates were washed and sequentially incubated with an oligoclonal pool of SARS2-2, SARS2-11, SARS2-16, SARS2-31, SARS2-38, SARS2-57, and SARS2-71^52^ anti-S antibodies and HRP-conjugated goat anti-mouse IgG (Sigma, 12-349) in PBS supplemented with 0.1% saponin and 0.1% bovine serum albumin. SARS-CoV-2-infected cell foci were visualized using TrueBlue peroxidase substrate (KPL) and quantitated on an ImmunoSpot microanalyzer (Cellular Technologies).

### Measurement of viral burden.

Tissues were weighed and homogenized with zirconia beads in a MagNA Lyser instrument (Roche Life Science) in 1000 μL of DMEM medium supplemented with 2% heat-inactivated FBS. Tissue homogenates were clarified by centrifugation at 10,000 rpm for 5 min and stored at −80°C. RNA was extracted using the MagMax mirVana Total RNA isolation kit (Thermo Fisher Scientific) on the Kingfisher Flex extraction robot (Thermo Fisher Scientific). RNA was reverse transcribed and amplified using the TaqMan RNA-to-CT 1-Step Kit (Thermo Fisher Scientific). Reverse transcription was carried out at 48°C for 15 min followed by 2 min at 95°C. Amplification was accomplished over 50 cycles as follows: 95°C for 15 s and 60°C for 1 min. Copies of SARS-CoV-2 N gene RNA in samples were determined using a previously published assay ^53^. Briefly, a TaqMan assay was designed to target a highly conserved region of the N gene (Forward primer: ATGCTGCAATCGTGCTACAA; Reverse primer: GACTGCCGCCTCTGCTC; Probe: /56-FAM/TCAAGGAAC/ZEN/AACATTGCCAA/3IABkFQ/). This region was included in an RNA standard to allow for copy number determination down to 10 copies per reaction. The reaction mixture contained final concentrations of primers and probe of 500 and 100 nM, respectively.

### Plaque assay.

Vero-TMPRSS2-hACE2 cells were seeded at a density of 1×10^5^ cells per well in 24-well tissue culture plates. The following day, medium was removed and replaced with 200 μL of material to be titrated diluted serially in DMEM supplemented with 2% FBS. One hour later, 1 mL of methylcellulose overlay was added. Plates were incubated for 72 h, then fixed with 4% paraformaldehyde (final concentration) in PBS for 20 min. Plates were stained with 0.05% (w/v) crystal violet in 20% methanol and washed twice with distilled, deionized water.

### Cytokine and chemokine protein measurements.

Lung homogenates were incubated with Triton-X-100 (1% final concentration) for 1 h at room temperature to inactivate SARS-CoV-2. Homogenates then were analyzed for cytokines and chemokines by Eve Technologies Corporation (Calgary, AB, Canada) using their Mouse Cytokine Array / Chemokine Array 31-Plex (MD31) platform.

### Data availability.

All data supporting the findings of this study are available within the paper and are available from the corresponding author upon request.

### Statistical analysis.

All statistical tests were performed as described in the indicated figure legends using Prism 8.0. Statistical significance was determined using an ordinary one-way ANOVA with Dunnett’s post-test when comparing three or more groups. The number of independent experiments used are indicated in the relevant Figure legends.

## Supplementary Material

Supplement 1

Supplement 2

Supplement 3

Supplement 4

Supplement 5

Supplement 6

Supplement 7

Supplement 8

Supplement 9

## Figures and Tables

**Table 1.** Neutralizing monoclonal antibodies

	Antibody	Class	Structural binding site (amino add position in spike protein)	Functional Residues	References
Vanderbilt University/AstraZeneca*	COV2-2130	RBM	345–346, 439–441, 443–447, 449–450, 452, 484, 490, 492–494	Mutational analysis/yeast display: R346, K444, G447, N448 VSV-SARS-CoV-2 escape: K444R/E Authentic SARS-CoV-2 escape: N74K, R346I	8,54,55
	COV2-2196	RBM	455–456, 475–479, 484–489, 493	Mutational analysis/yeast display: G476, F486, N487, Y489 VSV-SARS-CoV-2 escape: None identified Authentic SARS-CoV-2 escape: None identified	8,54,55
	COV2-2130/COV2-2196			Mutational analysis/yeast display: Not determined VSV-SARS-CoV-2: None identified Authentic SARS-CoV-2: None identified	55
Regeneron*	REGN10933	RBM	403, 406, 417, 421, 449, 453, 455-456, 473-478, 484-490, 492-496, 498, 501	Mutational analysis/yeast display: K417, Y453, L455, E484, G485, F486, N487, Y489, Q493 VSV-SARS-CoV-2 escape: K417E, Y453F, L455F, F486V, Q493K Authentic SARS-CoV-2 escape: F486I, Y489H, Q493K (identified in patients treated with REGN-COV2)	56,57
	REGN10987	RBM	346, 439-441, 443-450, 498-501	Mutational analysis/yeast display: N439, N440, K444, V445, G446, G447, N448, N439, N440, P499 VSV-SARS-CoV-2 escape: K444Q, V445A Authentic SARS-CoV-2 escape: N440D (identified in patients treated with REGN-COV2)	56,57
	REGN10933/REGN10987			Mutational Analysis/yeast display: E406W, Q493F VSV-SARS-CoV-2: None identified Authentic SARS-CoV-2: None identified	57
AbbVie	2B04	RBM	446, 449-450, 452, 455-456, 483-487, 489-490, 492-494, 496, 498	Mutational analysis/yeast display: Not determined VSV-SARS-CoV-2 escape: E484K/A, F486S Authentic SARS-CoV-2 escape: Not determined	52 Errico, Fremont et al., unpublished data
	47D11	RBM	338-339, 342-343, 345, 365, 367-368, 374, 436-437	Mutational Analysis/yeast display: F338, F342, N343, Y365, L368, F374, W436 VSV-SARS-CoV-2 escape: L335F/W/S, G339S/V/D, E340K, F338L, N434Y, Authentic SARS-CoV-2 escape: Not determined	https://www.biorxiv.Org/content/10.1101/2020.09.30.3 58 Liu, Whelan et al., unpublished data
	2B04/47D11			Mutational analysis/yeast display: Not determined VSV-SARS-CoV-2 escape: Not determined Authentic SARS-CoV-2 escape: Not determined	
Vir Biotechnology	S309	Base of RBD	333–335, 337, 339–341, 343, 346, 354, 356–361, 440–441, 444, 509	Mutational analysis/yeast display: P337, E340 VSV-SARS-CoV-2 escape: E340A/K/G, P337L Authentic SARS-CoV-2 escape: E340A	6,59
	S2E12	RBM	455–458, 473–493	Mutational analysis/yeast display: A475, G476, F486, N487,Y489 VSV-SARS-CoV-2 escape: A475D, G476D/S, G485D Authentic SARS-CoV-2 escape: Not determined	10,59 Liu, Whelan et al. unpublished data
	S309/S2E12			Mutational analysis/yeast display: Not determined VSV-SARS-CoV-2 escape: Not determined Authentic SARS-CoV-2 escape: Not determined	
Lilly*	LY-CoV555	RBM	434-444, 455-456, 484, 486-490, 493-494	Yeast display: L452, I472, V483, E848, G485, F486, F490, Q493, S494 VSV-SARS-CoV-2 escape: Not determined Authentic SARS-CoV-2 escape: E484K	2,36,60

* Corresponding antibody sequence

## References

[1] Chen R.E., Resistance of SARS-CoV-2 variants to neutralization by monoclonal and serum-derived polyclonal antibodies. Nat Med(2021). 10.1038/s41591-021-01294-w PMC8058618 33664494

[2] Wang P., Antibody Resistance of SARS-CoV-2 Variants B. 1.351 and B.1.1.7. Nature(2021). 10.1038/s41586-021-03398-2 33684923

[3] Wang Z., mRNA vaccine-elicited antibodies to SARS-CoV-2 and circulating variants. Nature(2021). 10.1038/s41586-021-03324-6 PMC8503938 33567448

[4] Sempowski G.D., Saunders K.O., Acharya P., Wiehe K.J. & Haynes B.F. Pandemic Preparedness: Developing Vaccines and Therapeutic Antibodies For COVID-19. Cell 181, 1458–1463 (2020). 32492407 10.1016/j.cell.2020.05.041 PMC7250787

[5] Letko M., Marzi A. & Munster V. Functional assessment of cell entry and receptor usage for SARS-CoV-2 and other lineage B betacoronaviruses. Nature microbiology 5, 562–569 (2020). 10.1038/s41564-020-0688-y PMC7095430 32094589

[6] Pinto D., Cross-neutralization of SARS-CoV-2 by a human monoclonal SARS-CoV antibody. Nature 583, 290–295 (2020). 32422645 10.1038/s41586-020-2349-y

[7] Cao Y., Potent neutralizing antibodies against SARS-CoV-2 identified by high-throughput single-cell sequencing of convalescent patients' B cells. Cell 182, 73–84(2020). 32425270 10.1016/j.cell.2020.05.025 PMC7231725

[8] Zost S.J., Rapid isolation and profiling of a diverse panel of human monoclonal antibodies targeting the SARS-CoV-2 spike protein. Nat Med 26, 1422–1427 (2020). 32651581 10.1038/s41591-020-0998-x PMC8194108

[9] Barnes C.O., SARS-CoV-2 neutralizing antibody structures inform therapeutic strategies. Nature 588, 682–687 (2020). 33045718 10.1038/s41586-020-2852-1 PMC8092461

[10] Tortorici M.A., Ultrapotent human antibodies protect against SARS-CoV-2 challenge via multiple mechanisms. Science 370, 950–957 (2020). 32972994 10.1126/science.abe3354 PMC7857395

[11] Rathe J.A., SARS-CoV-2 Serologic Assays in Control and Unknown Populations Demonstrate the Necessity of Virus Neutralization Testing. J Infect Dis (2020). 10.1093/infdis/jiaa797 PMC7798987 33367830

[12] Wibmer C.K., SARS-CoV-2 501Y.V2 escapes neutralization by South African COVID-19 donor plasma. bioRxiv(2021). 10.1038/s41591-021-01285-x 33654292

[13] Tada T., Neutralization of viruses with European, South African, and United States SARS-CoV-2 variant spike proteins by convalescent sera and BNT162b2 mRNA vaccine-elicited antibodies. bioRxiv(2021).

[14] Leung K., Shum M.H., Leung G.M., Lam T.T. & Wu J.T. Early transmissibility assessment of the N501Y mutant strains of SARS-CoV-2 in the United Kingdom, October to November 2020. Euro Surveill 26(2021). 10.2807/1560-7917.ES.2020.26.1.2002106 PMC7791602 33413740

[15] Xie X., Neutralization of SARS-CoV-2 spike 69/70 deletion, E484K and N501Y variants by BNT162b2 vaccine-elicited sera. Nat Med (2021). 10.1038/s41591-021-01270-4 33558724

[16] Klimstra W.B., SARS-CoV-2 growth, furin-cleavage-site adaptation and neutralization using serum from acutely infected hospitalized COVID-19 patients. J Gen Virol 101, 1156–1169 (2020). 32821033 10.1099/jgv.0.001481 PMC7879561

[17] Johnson B.A., Loss of furin cleavage site attenuates SARS-CoV-2 pathogenesis. Nature(2021). 10.1038/s41586-021-03237-4 PMC8175039 33494095

[18] Winkler E.S., SARS-CoV-2 infection of human ACE2-transgenic mice causes severe lung inflammation and impaired function. Nat Immunol 21, 1327–1335(2020). 32839612 10.1038/s41590-020-0778-2 PMC7578095

[19] McCray P.B. Jr., Lethal infection of K18-hACE2 mice infected with severe acute respiratory syndrome coronavirus. J Virol 81, 813–821 (2007). 17079315 10.1128/JVI.02012-06 PMC1797474

[20] Winkler E.S., Human neutralizing antibodies against SARS-CoV-2 require intact Fc effector functions for optimal therapeutic protection. Cell 184, 1804–1820.e1816 (2021). 33691139 10.1016/j.cell.2021.02.026 PMC7879018

[21] Rathnasinghe R., The N501Y mutation in SARS-CoV-2 spike leads to morbidity in obese and aged mice and is neutralized by convalescent and post-vaccination human sera. medRxiv: the preprint server for health sciences (2021).

[22] Gu H., Adaptation of SARS-CoV-2 in BALB/c mice for testing vaccine efficacy. Science 369, 1603–1607 (2020). 32732280 10.1126/science.abc4730 PMC7574913

[23] Oliphant T., Development of a humanized monoclonal antibody with therapeutic potential against West Nile virus. Nature Medicine 11, 522–530 (2005). 10.1038/nm1240 PMC1458527 15852016

[24] Giamarellos-Bourboulis E.J., Complex Immune Dysregulation in COVID-19 Patients with Severe Respiratory Failure. Cell Host Microbe (2020). 10.1016/j.chom.2020.04.009 PMC7172841 32320677

[25] Rosenfeld R., Post-exposure protection of SARS-CoV-2 lethal infected K18-hACE2 transgenic mice by neutralizing human monoclonal antibody. Nat Commun 12, 944 (2021). 33574228 10.1038/s41467-021-21239-8 PMC7878817

[26] García-Arriaza J., COVID-19 vaccine candidates based on modified vaccinia virus Ankara expressing the SARS-CoV-2 spike induce robust T- and B-cell immune responses and full efficacy in mice. J Virol(2021). 10.1128/JVI.02260-20 PMC8092708 33414159

[27] Zheng J., COVID-19 treatments and pathogenesis including anosmia in K18-hACE2 mice. Nature 589, 603–607 (2021). 33166988 10.1038/s41586-020-2943-z PMC7855185

[28] Hassan A.O., A Single-Dose Intranasal ChAd Vaccine Protects Upper and Lower Respiratory Tracts against SARS-CoV-2. Cell 183, 169–184.e113 (2020). 32931734 10.1016/j.cell.2020.08.026 PMC7437481

[29] Zhou D., Robust SARS-CoV-2 infection in nasal turbinates after treatment with systemic neutralizing antibodies. Cell Host Microbe (2021). 10.1016/j.chom.2021.02.019 PMC7904446 33657424

[30] Andreano E., Extremely potent human monoclonal antibodies from COVID-19 convalescent patients. Cell 184, 1821–1835.e1816 (2021). 33667349 10.1016/j.cell.2021.02.035 PMC7901298

[31] Cobey S., Larremore D.B., Grad Y.H. & Lipsitch M. Concerns about SARS-CoV-2 evolution should not hold back efforts to expand vaccination. Nat Rev Immunol, 1–6 (2021). 33795856 10.1038/s41577-021-00544-9 PMC8014893

[32] Shen X., Neutralization of SARS-CoV-2 Variants B.1.429 and B.1.351. N Engl J Med (2021). 10.1056/NEJMc2103740 PMC8063884 33826819

[33] McCallum M., SARS-CoV-2 immune evasion by variant B.1.427/B.1.429. bioRxiv(2021). 10.1126/science.abi7994 PMC9835956 34210893

[34] Lundgren J.D., A Neutralizing Monoclonal Antibody for Hospitalized Patients with Covid-19. N Engl J Med 384, 905–914 (2021). 33356051 10.1056/NEJMoa2033130 PMC7781100

[35] Gottlieb R.L., Effect of Bamlanivimab as Monotherapy or in Combination With Etesevimab on Viral Load in Patients With Mild to Moderate COVID-19: A Randomized Clinical Trial. Jama 325, 632–644 (2021). 33475701 10.1001/jama.2021.0202 PMC7821080

[36] Starr T.N., Greaney A.J., Dingens A.S. & Bloom J.D. Complete map of SARS-CoV-2 RBD mutations that escape the monoclonal antibody LY-CoV555 and its cocktail with LY-C0VOI6. Cell reports. Medicine, 100255 (2021). 33842902 10.1016/j.xcrm.2021.100255 PMC8020059

[37] Schmitz A.J., A public vaccine-induced human antibody protects against SARS-CoV-2 and emerging variants. bioRxiv(2021). 10.1016/j.immuni.2021.08.013 PMC8367776 34464596

[38] Tian F., Mutation N501Y in RBD of Spike Protein Strengthens the Interaction between COVID-19 and its Receptor ACE2. BioRxiv(2021).

[39] Bailey A.L. & Diamond M.S. A Crisp(r) New Perspective on SARS-CoV-2 Biology. Cell 184, 15–17 (2021). 33338422 10.1016/j.cell.2020.12.003 PMC7746090

[40] Pierson T.C. & Diamond M.S. A game of numbers: the stoichiometry of antibody-mediated neutralization of flavivirus infection. Progress in molecular biology and translational science 129, 141–166 (2015). 25595803 10.1016/bs.pmbts.2014.10.005 PMC4910618

[41] Graham C., Neutralization potency of monoclonal antibodies recognizing dominant and subdominant epitopes on SARS-CoV-2 Spike is impacted by the B.1.1.7 variant. Immunity(2021). 10.1016/j.immuni.2021.03.023 PMC8015430 33836142

[42] Rees-Spear C., The effect of spike mutations on SARS-CoV-2 neutralization. Cell Rep 34, 108890 (2021). 33713594 10.1016/j.celrep.2021.108890 PMC7936541

[43] Zhou D., Evidence of escape of SARS-CoV-2 variant B.1.351 from natural and vaccine-induced sera. Cell(2021). 10.1016/j.cell.2021.02.037 PMC7901269 33730597

[44] Baum A., Antibody cocktail to SARS-CoV-2 spike protein prevents rapid mutational escape seen with individual antibodies. Science(2020). 10.1126/science.abd0831 PMC7299283 32540904

[45] Goddard T.D., UCSF ChimeraX: Meeting modern challenges in visualization and analysis. Protein Sci 27, 14–25 (2018). 28710774 10.1002/pro.3235 PMC5734306

[46] Zang R., TMPRSS2 and TMPRSS4 promote SARS-CoV-2 infection of human small intestinal enterocytes. Sci Immunol 5(2020). 10.1126/sciimmunol.abc3582 PMC7285829 32404436

[47] Plante J.A., Spike mutation D614G alters SARS-CoV-2 fitness. Nature(2020). 10.1038/s41586-020-2895-3 PMC8158177 33106671

[48] Alsoussi W.B., A Potently Neutralizing Antibody Protects Mice against SARS-CoV-2 Infection. J Immunol (2020). 10.4049/jimmunol.2000583 PMC7566074 32591393

[49] Baum A., REGN-COV2 antibodies prevent and treat SARS-CoV-2 infection in rhesus macaques and hamsters. Science (2020). 10.1126/science.abe2402 PMC7857396 33037066

[50] Jones B.E., LY-CoV555, a rapidly isolated potent neutralizing antibody, provides protection in a non-human primate model of SARS-CoV-2 infection. bioRxiv(2020).

[51] Shi R., A human neutralizing antibody targets the receptor-binding site of SARS-CoV-2. Nature 584, 120–124 (2020). 32454512 10.1038/s41586-020-2381-y

[52] Liu Z., Identification of SARS-CoV-2 spike mutations that attenuate monoclonal and serum antibody neutralization. Cell Host Microbe 29, 477–488.e474 (2021). 33535027 10.1016/j.chom.2021.01.014 PMC7839837

[53] Case J.B., Bailey A.L., Kim A.S., Chen R.E. & Diamond M.S. Growth, detection, quantification, and inactivation of SARS-CoV-2. Virology 548, 39–48 (2020). 32838945 10.1016/j.virol.2020.05.015 PMC7293183

[54] Zost S.J., Potently neutralizing and protective human antibodies against SARS-CoV-2. Nature 584, 443–449 (2020). 32668443 10.1038/s41586-020-2548-6 PMC7584396

[55] Dong J., Genetic and structural basis for recognition of SARS-CoV-2 spike protein by a two-antibody cocktail. bioRxiv(2021).

[56] Hansen J., Studies in humanized mice and convalescent humans yield a SARS-CoV-2 antibody cocktail. Science 369, 1010–1014 (2020). 32540901 10.1126/science.abd0827 PMC7299284

[57] Starr T.N., Prospective mapping of viral mutations that escape antibodies used to treat COVID-19. bioRxiv(2020). 10.1126/science.abf9302 PMC7963219 33495308

[58] Wang C., A human monoclonal antibody blocking SARS-CoV-2 infection. Nat Commun 11, 2251 (2020). 32366817 10.1038/s41467-020-16256-y PMC7198537

[59] Starr T.N., Antibodies to the SARS-CoV-2 receptor-binding domain that maximize breadth and resistance to viral escape. bioRxiv(2021).

[60] Jones B.E., The neutralizing antibody, LY-CoV555, protects against SARS-CoV-2 infection in non-human primates. Sci Transl Med(2021). 10.1126/scitranslmed.abf1906 PMC8284311 33820835

