# Supplementary figures and images for: In vivo monoclonal antibody efficacy against SARS-CoV-2 variant strains
Source: Res Sq. 2021 Apr 23:rs.3.rs-448370. Preprint. [Version 1] doi: 10.21203/rs.3.rs-448370/v1 (PMC8132254; doi:10.21203/rs.3.rs-448370/v1)

2B04

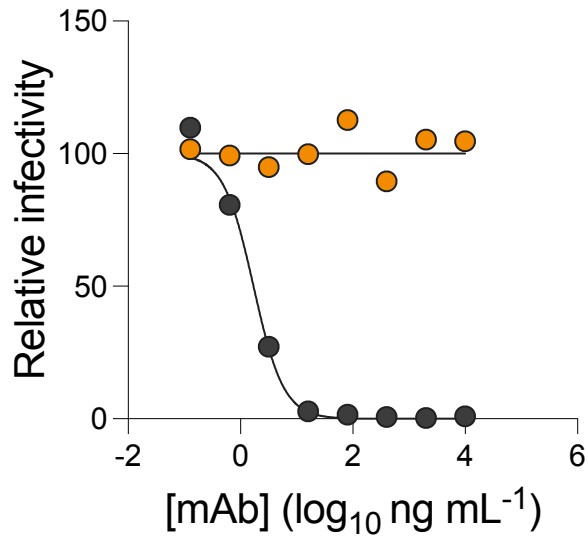

LY-CoV555

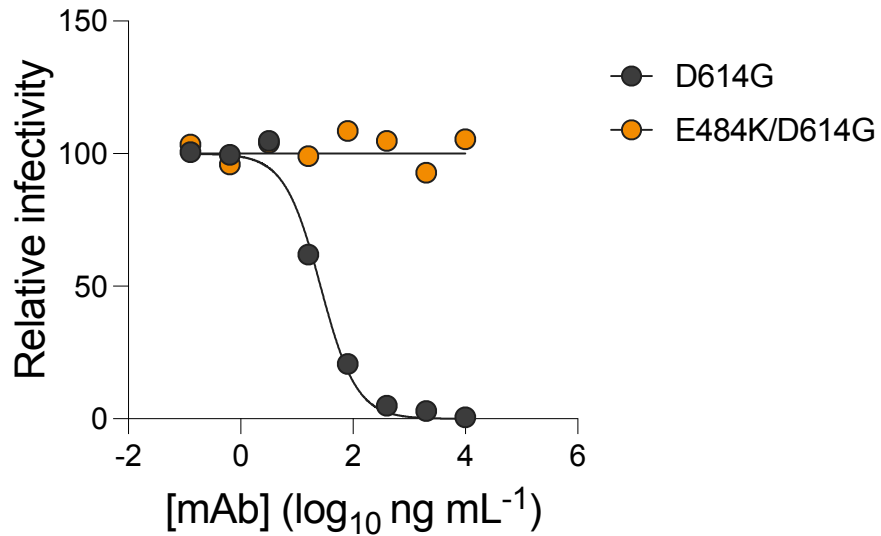

Extended Data Figure 1

Supplement: Supplement 1 [file 7d9c080c87ed199aa396005d.pdf]

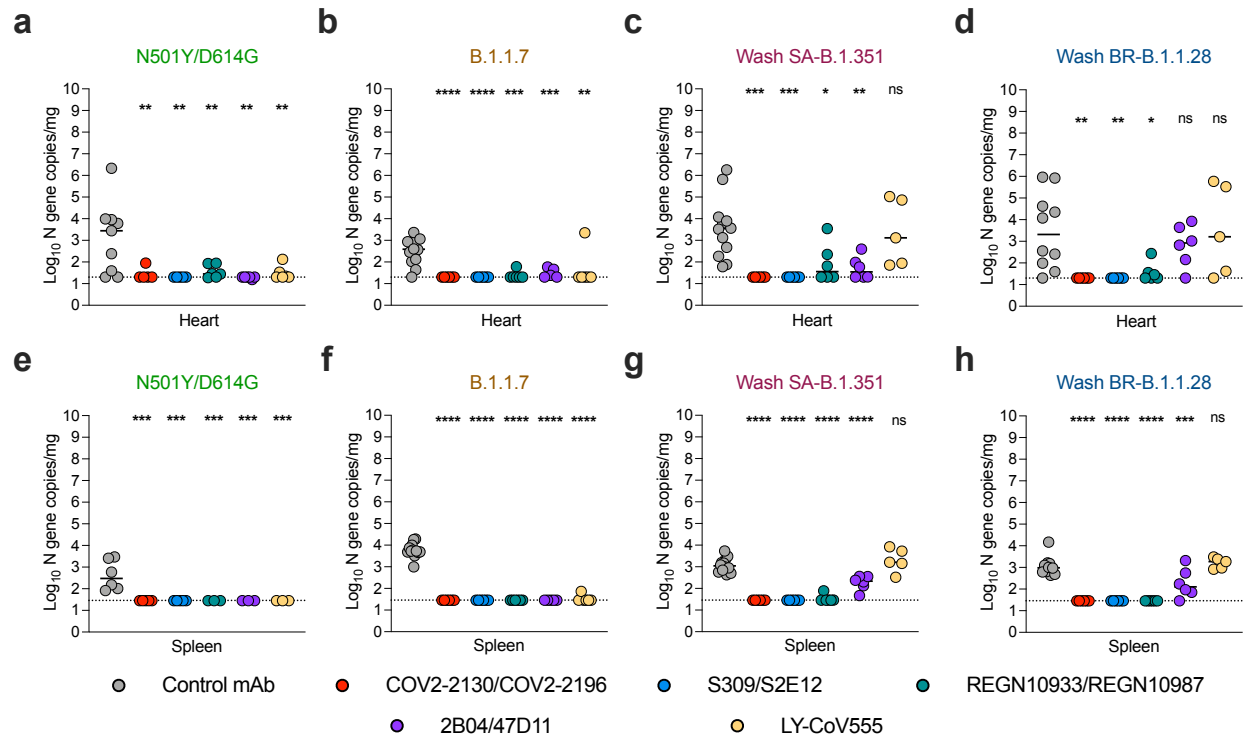

**Extended Data Figure 2**

Supplement: Supplement 2 [file 5ae87761f5c4afc8402df11a.pdf]

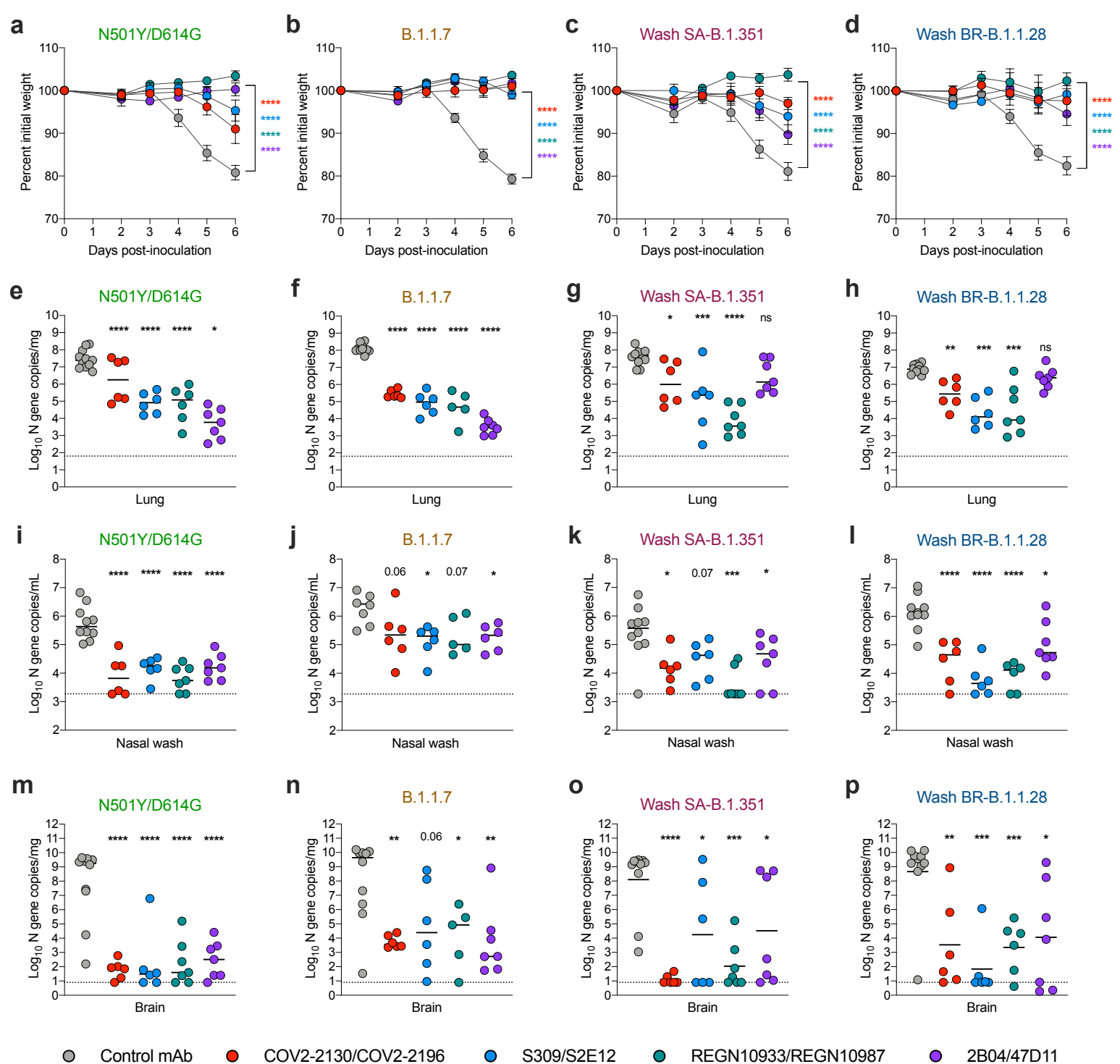

Extended Data Figure 5

Supplement: Supplement 5 [file b530e73c372784e777262c15.pdf]

# CB6

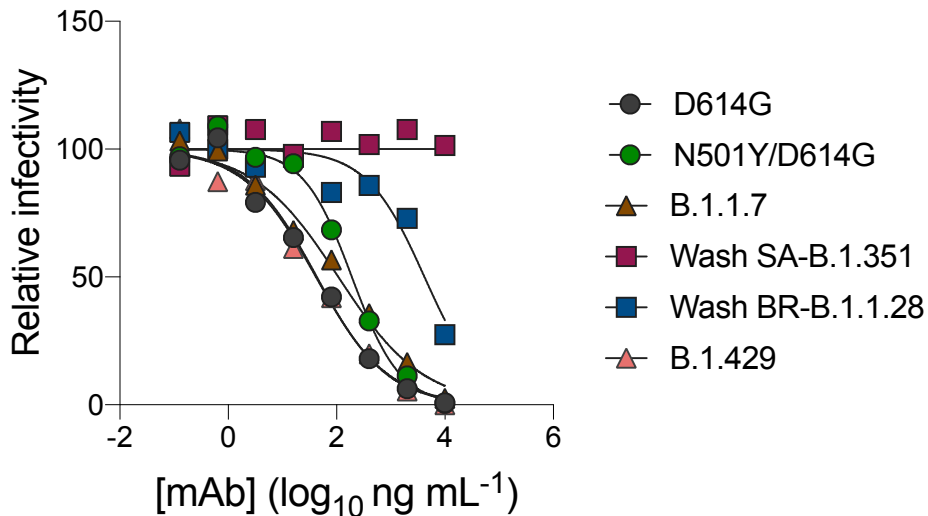

**Extended Data Figure 6**

Supplement: Supplement 6 [file 1659faaad84dafac7c8151f4.pdf]
