# Supplementary material for: In vivo monoclonal antibody efficacy against SARS-CoV-2 variant strains
Source: Res Sq. 2021 Apr 23:rs.3.rs-448370. Preprint. [Version 1] doi: 10.21203/rs.3.rs-448370/v1 (PMC8132254; doi:10.21203/rs.3.rs-448370/v1)
Supplement: Supplement 3 [file 7a180a30d5aa7eb35ad787af.pdf]

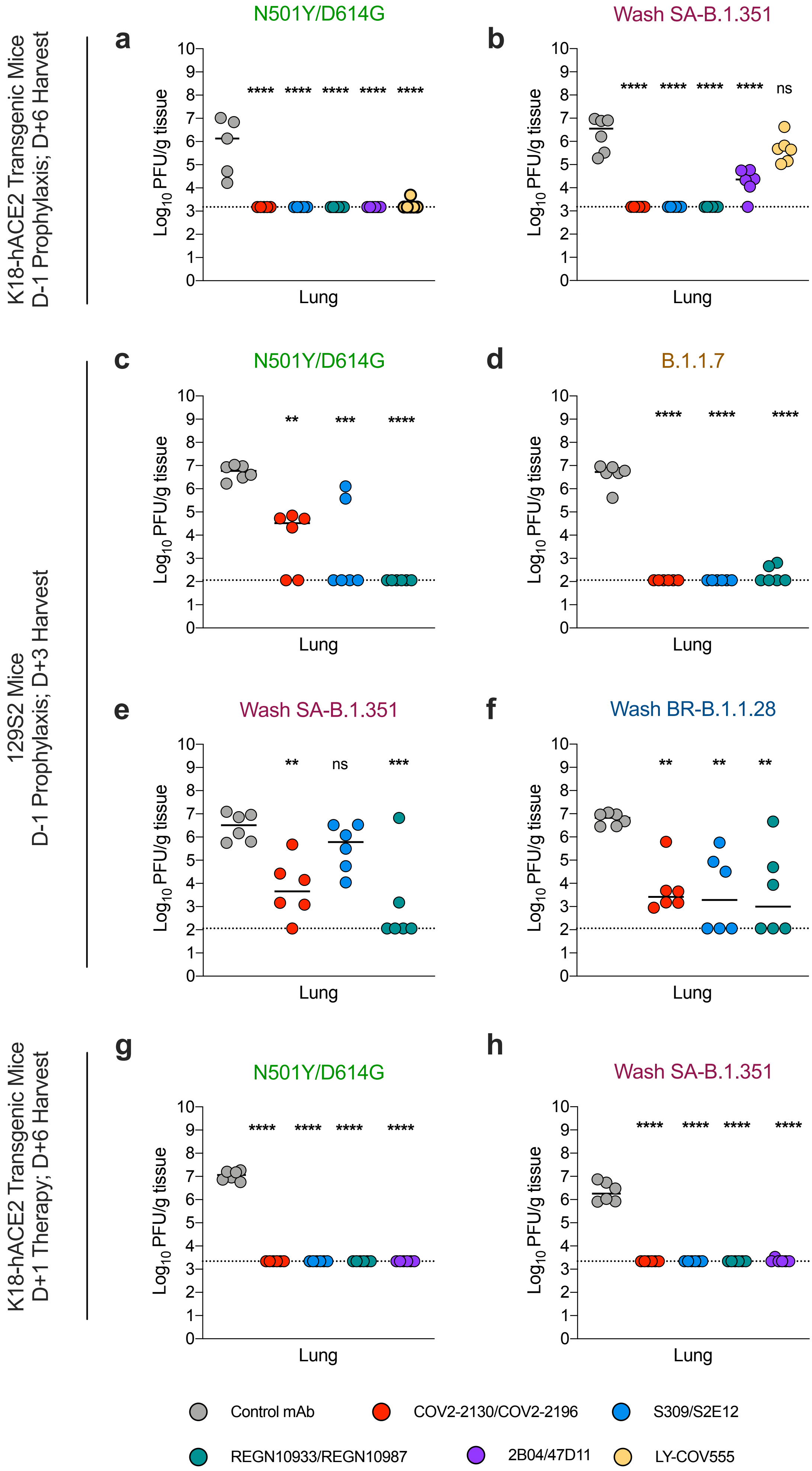

Extended Data Figure 3
